# Dr. Samuel Houston

**Published:** 1908-01

**Authors:** 


Dr. Samuel Houston, of Washington, D. C., medical referee of the bureau of pensions, died at Garfield Hospital, November 17, 1907, aged 65 years. He graduated at Georgetown University

School of Medicine in 1868, and had been connected with the U. S. Pension bureau in an official capacity for more than twenty-five years. He was a capable officer and enjoyed the confidence of his professional and official colleagues.

